# A Study of the Influence of Cement Addition and Humidity on the Mechanical Strength and Microstructure of Flue Gas Desulfurization Gypsum–Cement Plasters

**DOI:** 10.3390/ma17102374

**Published:** 2024-05-15

**Authors:** Edyta Baran, Mariusz Hynowski, Łukasz Kotwica, Jacek Rogowski

**Affiliations:** 1 Research and Development Center, Branch Leszcze, Atlas sp. z o.o., Leszcze 15, 28-400 Pińczów, Poland; mhynowski@atlas.com.pl; 2 Department of Building Materials Technology, Faculty of Materials Science and Ceramics, AGH University of Krakow, al. Adama Mickiewicza 30, 30-059 Kraków, Poland; lkotwica@agh.edu.pl; 3 Institute of General and Ecological Chemistry, Łódź University of Technology, ul. Żeromskiego 116, 90-924 Łódź, Poland; jacek.rogowski@p.lodz.pl

**Keywords:** FGD gypsum, Portland cement, mechanical strength, porosity, microstructure

## Abstract

Over the last 20 years, flue gas desulfurization gypsum (FGD gypsum) has become a valuable and widely used substitute for a natural raw material to produce plasters, mortars, and many other construction products. The essential advantages of FGD gypsum include its high purity and stability, which allow for better technical parameters compared to natural gypsum, and, until recently, its low price and easy availability. This FGD gypsum is obtained in the process of desulfurization of flue gases and waste gases in power plants, thermal power plants, refineries, etc., using fossil fuels such as coal or oil. The gradual reduction in energy production from fossil raw materials implemented by European Union countries until its complete cessation in 2049 in favor of renewable energy sources significantly affects the availability of synthetic gypsum, and forces producers of mortars and other construction products to look for new solutions. The gypsum content in commonly used light plaster mortars is usually from 50 to 60% by mass. This work presents the results of tests on mortars wherein the authors reduced the amount of gypsum to 30%, and, to meet the strength requirements specified in the EN 13279-1:2008 standard, added Portland cement in the amount of 6–12% by mass. Such a significant reduction in the content of synthetic gypsum will reduce this raw material’s consumption, thus extending its availability and developing other solutions. The study presented the test results on strength, density, porosity, pore size distribution, and changes in the microstructure of mortars during up to 180 days of maturation in conditions of increased relative humidity. The results show that decreased porosity and increased mechanical strength occur due to the densification of the microstructure caused by the formation of hydration products, such as C-S-H, ettringite, and thaumasite.

## 1. Introduction

Calcium sulfate dihydrate, obtained as a by-product in desulfurizing waste gases in power plants, refineries, and thermal power plants, is a valuable source of construction gypsum. Compared to natural gypsum, the advantage of FGD gypsum is the high calcium sulfate content exceeding 95%, which translates into the mechanical strength of the gypsum material, which is 35–40% higher [1]. Moreover, the lack of contamination with clay minerals, limestone, and silica, homogeneity, high availability, and relatively low price, compared to natural gypsum, contributed to greater interest in using this material. In Poland, since 1994, the use of natural gypsum in plasters has been replaced by FGD gypsum, and there has been a significant increase in FGD gypsum production since 2008. In addition to the advantages of FGD gypsum, investments were made to reduce SO_2_ emissions into the atmosphere [2]. The high operating costs of quarries extracting natural gypsum stone influenced this situation. It is worth emphasizing that plaster that is made based on FGD gypsum is characterized by more excellent stability, uniformity, and more straightforward application, which is essential from the contractors’ point of view. Gypsum is used to produce construction products such as plasters, mortars, and prefabricated elements [3] and as a setting regulator in cement [4,5,6].

The changes currently taking place in the energy sector aimed at reducing greenhouse gas emissions, involve abandoning fossil fuels, including hard coal and lignite, which are the basis for energy production in Poland and result in FGD gypsum as a by-product. The ongoing transformations are related to implementing the National Energy and Climate Plan for 2021–2030, which, in particular, assumes a reduction to 50–60% of the share of coal in electricity production [7]. Poland has scheduled to end energy production based on hard coal and lignite by 2049 [8,9]. This means a substantial reduction in FGD production. As a result of the introduced regulations, the production of FGD gypsum is and will be decreasing every year [10], which results in problems with the availability and stability of products produced so far, including plasters.

The decreasing availability of FGD gypsum, along with its anticipated scarcity in the future, compels manufacturers of building mortars to seek alternative solutions. One of the rational transitional solutions that extend the availability of gypsum-based construction products is reducing the share of the gypsum binder in plasters, which currently amounts to 55–65%. The EN 13279-1:2008 standard [11] requires plasters in categories B1 (gypsum plaster), B4 (light gypsum plaster), B7 (gypsum plaster with increased surface hardness), and C6 (thin-layer gypsum plaster) to contain at least 50% gypsum binder. These are the most frequently produced gypsum plasters in Poland and Europe. The high gypsum binder content in plasters results from the need to meet the strength requirements of the standard [11], according to which the bending strength for plasters of categories B1, B4, and C6 is above 1.0 MPa, and above 2.0 MPa for plasters of category B7, and the compressive strength for plasters of categories B1, B4, and C6 is above 2.0 MPa, and is above 6.0 MPa for plasters of category B7. The share of gypsum binder above 50% affects high efficiency, ease of application, and low product density. The standard [11] allows the use of gypsum plasters with a reduced content of gypsum binder (below 50%), defined as plasters of categories B2 (gypsum-based plaster) and B5 (light gypsum-based plasters). However, they are subject to the exact strength requirements as plasters of categories B1 and B4 (containing at least 50% same gypsum binder), which poses a challenge for plaster producers in terms of maintaining the required technical parameters while maintaining current standards of application, quality, and efficiency. One way to solve this problem may be to replace even half of the commonly used amount of gypsum binder with another binder, such as Portland cement. The addition of cement will make it possible to achieve the required strength parameters while taking advantage of the relatively low content of FGD gypsum in the composition of mortars. Moreover, a plaster with a mixture of these two binders with a relatively small amount of Portland cement (6–8%) also meets strength requirements for internal cement plastering mortars (category CSII, CSIII), for which its content is usually higher (10–20%). That will be more beneficial for the environment in the context of binder savings and the issue of CO_2_ emissions accompanying cement production.

Over the years, researchers have attempted to develop building materials with a smaller amount of gypsum binder. The main goal of some of these works was to increase their usefulness as construction materials by increasing water resistance [12,13]. The best improvement in water resistance, and thus better mechanical strength results, was obtained for gypsum–cement–gypsum–cementpozzolanic mixtures [14,15,16,17,18]. The presence of the calcium silicates hydrates (C-S-H phase) [19] formed as a result of the hydrolysis of calcium silicates from cement and additional amounts of the C-S-H phase formed as a result of the pozzolanic reaction led to a decrease in porosity and a shift in pore size towards smaller ones in the process of pore refinement. Test results for gypsum–cement gypsum–cementcomposites without hydraulic and pozzolanic additives stored in water for more than 28 days indicate that these composites deteriorated under these conditions. That confirms that this type of material is not water-resistant [20,21,22,23]. The full moisture composite caused the dissolution of dihydrate gypsum crystals, weakening the composite microstructure, reducing strength, and increasing porosity. The above limitations thus narrowed the scope of their application. However, it is important to emphasize that the pore refinement in the case of composites made of mineral binders leads to a decrease in permeability, and thus, improves the material durability in a corrosive environment. That is especially important from the point of view of sustainability. The more durable material will last longer and, thus, have an enhanced service life. That is the reason why durability, which is designed, among others, by proper porosity structure, should be taken into account during life-cycle assessment [24].

The authors of the study [25] showed that it is possible to develop plasters containing approximately half as much gypsum binder, i.e., 30% by mass, which will also meet the requirements of the standard [11] thanks to the addition of Portland cement in the amount of 6–12% by mass. Despite the confirmed formation of expansive sulfate-clay and sulfate-carbonate-silicate phases in such plasters, their presence did not negatively affect the mechanical strength of the prepared gypsum–cement system [20,25].

In addition to the chemical composition, another critical factor influencing the mechanical strength of gypsum–cement mortars is their total porosity, pore size, shape, and limiting pore diameter. The relationship between these parameters, and mechanical strength and durability has been previously investigated for cement pastes [26,27,28,29,30]. Mindess [31] showed that the greater the share of fine pores and the smaller the average pore size for a given material porosity, the greater its strength. Increasing the amount of mineral additives in cement systems, such as blast furnace slag [32] or pozzolans [33,34], reduces capillary porosity by filling the pores and significantly reduces the dimensions of larger pores. Pandey et al. [29] studied the effect of various mineral additives on the porosity and strength of cement mortars on the 7th, 28th, and 90th day of hydration. The modified cement mortar had a higher porosity than the mortar without additives. Additionally, observers noted a decrease in porosity as the hydration process progressed, which they explained by the gradual filling of large pores with hydration products, such as ettringite, portlandite, C-S-H, and others. Similar conclusions were reached by the authors of other publications [26,35], indicating a decrease in mechanical strength with an increase in mortar porosity. The shape and size of the solid phase crystallites also significantly impact the porosity and strength of the material [36]. The improvement of the mechanical properties of cement composites in the initial phase of exposure to sulfate solutions resulted from the crystallization of ettringite in the material pores, leading to the sealing of the microstructure and a decrease in porosity [37].

In the case of gypsum mortars, the porosity and pore size distribution depend mainly on the amount of mixing water, which translates into the material’s mechanical strength. Studies have shown that the higher the water content in the system, the higher the porosity of the gypsum material and the greater the number of pores with larger diameters. The hardened gypsum slurry has an unimodal pore size distribution, with the most significant number of pores in the 1–10 µm range [38]. However, in gypsum mortar, pores occupy approximately 50–60% of the volume, and a significant number have diameters in the range of 0.3–1.0 µm [39]. The origin and grain structure of the filler used, include crushed rock aggregate, uncrushed sand from the sand pit, and uncrushed river sand [40]. In gypsum mortars with the addition of aggregates with a smooth surface, pores with a diameter range of 0.1–10.0 µm predominate, while the use of aggregates with a rough surface shifts the maximum peak in the pore distribution towards pores with smaller diameters, i.e., 0.1–1.0 µm. In gypsum mortars, the presence of pores with a diameter larger than 10 µm results from the large amount of water in the paste [41]. Freire et al. [42] examined changes in the porosity of gypsum and gypsum-lime mixtures after 90 days and two years. The publication’s authors observed a decrease in porosity over time for products with the addition of lime by approximately 10%, which was attributed to the carbonation process. Another study [43] found the porosity of gypsum-lime mortars to range from 42 to 57%.

This paper presents the results of testing gypsum–cement plasters containing a reduced amount of gypsum binder (30% by mass) intended for finishing works inside buildings. The main aim of the research was to analyze the porosity of gypsum–cement plasters exposed to increased relative humidity for 180 days and to determine its impact on the mechanical strength of the mortars. The suitability of gypsum–cement plasters for use in rooms with increased relative humidity, such as bathrooms, laundry rooms, and kitchens, can be practically assessed using the obtained results. In these environments, there is a risk of adverse effects of moisture on their physical and mechanical properties, especially over an extended period of exposure.

## 2. Materials and Methods

### 2.1. Materials

**Figure 1:**
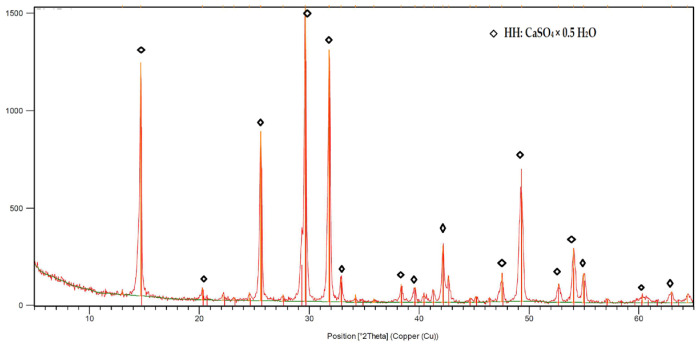
XRD analysis of FGD gypsum (β-hemihydrate) obtained from the Kozienice Power Plant.

**Figure 2:**
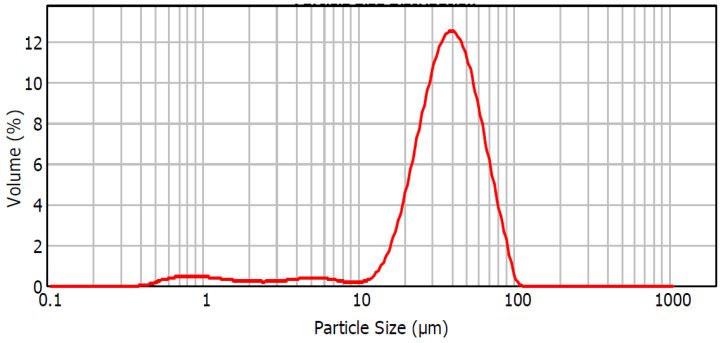
Particle size distribution of FGD gypsum (β-hemihydrate) obtained from the Kozienice Power Plant.

Four types of plasters were prepared, containing 30% by mass of FGD gypsum (d_50_ = 37.8 µm) obtained from the Kozienice Power Plant (Świerże Górne, Poland). The X-ray diffraction (XRD, Philips PW 1030) pattern of FGD gypsum (β-hemihydrate) is shown in Figure 1, and the particle size distribution is shown in Figure 2. Portland Cement CEM I 42.5R from the Lafarge Kujawy Cement Plant was added to each plaster (chemical composition: 19.94% SiO_2_, 5.14% Al_2_O_3_, 2.89% Fe_2_O_3_, 63.63% CaO, 1.4% MgO, 3.17% SO_3_, 0.6% K_2_O, 0.2% Na_2_O, 0.074% Cl^−^, 0.12% P_2_O_5_) in amounts of 6%, 8%, 10% and 12% by mass.

Table 1 contains the compositions of the prepared plasters, described according to the XG/YC scheme, where X—is the percentage of gypsum (30%), Y—is the percentage of cement (6%, 8%, 10%, 12%), G means gypsum, and C means cement.

The fillers used were quartz sand, limestone flour, and expanded perlite. To improve plasticity and workability, hydrated lime and chemical admixtures were used in the form of modified methylcellulose (viscosity 35,000 mPa∙s), starch ether (viscosity 1000 mPa∙s), sodium lauryl sulfate (air entraining agent), and L (+) tartaric acid (setting time regulator). All plaster ingredients are available on the market and commonly used in industrial production.

### 2.2. Methods

#### 2.2.1. Preparation and Storage of Gypsum–Cement Plaster Samples for Testing

The consistency of four gypsum–cement plasters was determined for a flow diameter of (160 ± 5) mm using a shake table under the EN 13279-2:2014 standard [44]. Each plaster’s water–gypsum ratio (*w*/*g*) was constant and amounted to 0.45. Then, bars with dimensions of 4 × 4 × 16 cm were prepared and stored in a climatic chamber automatically monitored for 180 days at a temperature of (20 ± 2) °C and relative humidity (>95%). Hydration was stopped by drying the samples in a vacuum oven at 45 °C until the pressure stabilized at 100 mtorr. Then, samples were closed in vacuum-sealed plastic bags and stored under constant temperature conditions (20 ± 2 °C).

#### 2.2.2. Test Methods for Gypsum–Cement Plasters

##### Flexural and Compression Strength

The mechanical strength test was performed according to [44] using the Toni Technik Tonitrol 0510 testing machine. The load for flexural tests was increased uniformly in the range of 10 to 50 N/s, such that specimen fracture occurred from 30 s to 90 s. The load for compression tests was increased continuously at a rate in the range of 50 to 500 N/s until the failure of the specimen at a time from 30 s to 90 s. The determination of flexural strength was performed on three beams with dimensions of 160 × 40 × 40 mm, and the result is the arithmetic mean of the three values obtained, whereas, the compressive strength was applied to the halves of the beams previously subjected to bending, and the result is the arithmetic mean of the six measurements obtained. This paper presents the results after 7, 28, and 180 conditioning days.

##### Specific Density, Bulk Density, and Porosity

The specific density test was conducted on samples of irregular shape, weighing approximately 40–120 g, using a Quantachrome Ultrapyc 1200e helium pycnometer. The measurement was taken automatically, and the determination result is the arithmetic mean of five density measurements performed one after the other under constant assumptions and the same temperature conditions.

The bulk density test was performed using a hydrostatic balance. The test result is the arithmetic mean of three independent measurements. Plaster samples with regular shapes were soaked to 1/3 of their height; after 24 h, they were poured to 2/3 of their height, and after another 24 h, they were completely poured and then stored until the mass stabilized.

Porosity was calculated based on the obtained values of bulk density and specific density according to the following formula:

(1) $$P=\left(1-\frac{\rho_{0}}{\rho_{w}}\right)\cdot 100\%$$

$\rho_{0}$—bulk density, g/cm^3^

$\rho_{w}$—specific density, g/cm^3^

This paper presents the results after 7, 28, and 180 conditioning days.

##### Pore Size Distribution

The tests were performed using a PoreMaster 60 mercury porosimeter from Quantachrome Instruments with a pressure range from 3.5 kPa to 220 MPa. The measurement was performed on beam-shaped samples with dimensions 8 mm × 8 mm × 20 mm. This paper presents the results after 28 and 180 days of seasoning. The equivalent pore diameters were calculated using the Washburn equation, assuming the contact angle of the plaster with mercury was equal to 140° and the surface tension of the mercury–air interface was 0.485 N/m.

##### Analysis of Microstructure Changes

The studies used the FEI NanoSEM 200 scanning electron microscope in a low vacuum in the secondary electron (SE) mode (residual water vapor pressure of 60 Pa). Before observations, the samples were coated with a thin carbon layer to prevent charges from accumulating on the sample surface during testing. Microstructure images were taken for all mortars after 7, 28, and 180 days of conditioning. Additionally, EDS analysis of the 30G/10C sample was performed on the 180th day of seasoning.

##### XRD Analysis

Powder X-ray diffraction analysis was performed using a Philips PW 1030 diffractometer operating with CuKα radiation. XRD data were collected in the range 5–65° 2θ with a step of 0.05° and an exposition per one step of 1 s. The XRD patterns were collected for gypsum–cement mortar samples cured for 28 and 180 days at increased RH (>95%). Before the test, the samples were ground into powder and than passed through a 63 µm sieve.

##### Statistical Analysis

Flexural strength and bulk density were each measured at least three times (n = 3 individual measurements). Compressive strength was measured at least six times (n = 6 individual measurements). Specific density was measured at least five times (n = 5 individual measurements). The results are presented as the mean and standard deviation. Student’s test was employed to compare the measured values with the reference sample assuming equal variances, utilizing the null hypothesis (H0), which posits that the averages under investigation are equal (µ = µ0), with a probability determined by a given confidence level. In this study, the reference mean (µ0) was consistently the mean of the sample containing 6% mas. of cement. Level of significance was presented as * (*p* ≤ 0.05), ** (*p* ≤ 0.01), *** (*p* ≤ 0.001).

## 3. Results and Discussion

### 3.1. The Influence of Cement Addition on the Mechanical Strength of Gypsum–Cement Plasters

Table 2 contains the density, porosity, and mechanical strength test results. The specific densities of gypsum–cement plasters in the first days of the study ranged from 2.43 to 2.47 g/cm^3^. After six months, they went from 2.29 to 2.43 g/cm^3^. It means, that the specific density of the skeleton of the material is in general decreasing with time. This is because cement particles, the densest parts of the matrix, underwent hydration, and C-S-H phase together with other hydration products of lower specific densities formed. Increased bound water content results in increasing bulk density, and finally, due to the increased volume of solid parts of the material, its porosity is decreasing, as seen in Table 2.

**Figure 3:**
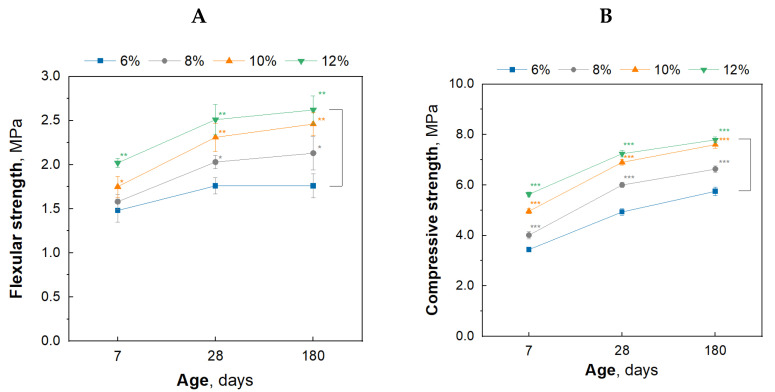
Effect of cement addition (6, 8, 10, and 12%) on the flexural (**A**) and compressive (**B**) strength of gypsum–cement plasters on the 7, 28, and 180 days of conditioning in increased relative humidity (>95%); Level of significance: * (*p* ≤ 0.05), ** (*p* ≤ 0.01), *** (*p* ≤ 0.001).

Additionally, the results of testing the mechanical strength of plasters containing different amounts of cement after various conditioning times are presented graphically in Figure 3.

The results presented in Table 2 and Figure 3 indicate that the flexural and compressive strength of the plasters increase with the addition of cement. In all plaster types, the most significant increase in flexural and compressive strength occurs between the 7th and 28th day of conditioning, followed by a slower rate of strength increase. The exception is sample 30G/6C, whose flexural strength does not change between 28 and 180 days of conditioning. This is because sample 30G/12C contains the least amount of cement and has the lowest mechanical strength parameters. The results suggest a greater degree of cement hydration in the first 28 days, then slower in the subsequent days of seasoning of the specimens under higher relative humidity conditions. The rate of strength increase in the initial conditioning period is related to the formation of gypsum hydration products and the formation of the C-S-H phase, resulting in the hydration of silicate cement phases [36].

The increase in mechanical strength that occurs for most plasters (Figure 3) between 28 and 180 days may be attributed to the formation of ettringite, thaumasite, or a mixture of ettringite and thaumasite crystals (woodfordite). The authors’ previous work described the formation of these phases in gypsum–cement plasters in conditions of increased humidity [25]. The possibility of a beneficial effect of the above stages on the mechanical strength of the tested mortars is consistent with the research results of Tesch and Middendorf [45], who showed that a small amount of formed ettringite or thaumasite did not result in deterioration of the mechanical properties and durability of gypsum-lime mortars.

### 3.2. The Influence of Cement Addition on the Bulk Density and Porosity of Gypsum–Cement Plasters

**Figure 4:**
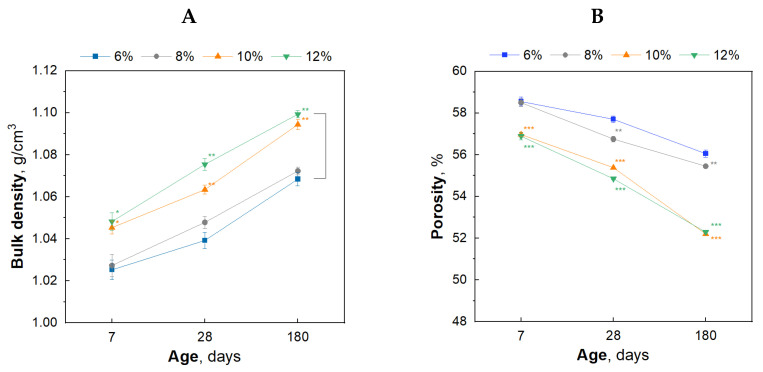
The effect of cement addition on the bulk density (**A**) and porosity (**B**) of gypsum–cement plasters on the 7th, 28th, and 180th day of conditioning in increased relative humidity (>95%); Level of significance: * (*p* ≤ 0.05), ** (*p* ≤ 0.01), *** (*p* ≤ 0.001).

The densities and porosities of mortars depending on the amount of added cement over 180 days in increased humidity are presented in Table 2 and graphically in Figure 4A,B. It is visible that the bulk density changes with increasing cement addition (Figure 4A). Moreover, an increase in density is observed during seasoning, i.e., with the progressive hydration process of plasters. The lowest porosity in the tested seasoning period was demonstrated by plasters with the highest cement content, i.e., 10 and 12% (Figure 4B). At the same time, for plasters with 6, 8, 10 and 12% cement content, a decrease in porosity was found between the 7th and 180th day of seasoning, by 2.5%, 3.1%, 4.8%, and 4.6%, respectively. At higher cement content, 10–12% *w*/*w* a decrease in porosity values are comparable. That is due to the high degree of the microstructure compaction. The most significant differences are between mortar samples containing less than 10% *w*/*w* cement. Based on the test results, the bulk density increases, and the porosity decreases with the increase in the cement content in the plasters and the progressing hydration process. The observed decrease in the porosity of gypsum–cement plasters may be caused by the filling of the pores and spaces between the gypsum crystals by the formed hydration products (e.g., C-S-H phase, ettringite, thaumasite), causing a reduction in the number of voids in the gypsum plaster [16].

### 3.3. Compressive Strength and Porosity of Gypsum–Cement Mortars

**Figure 5:**
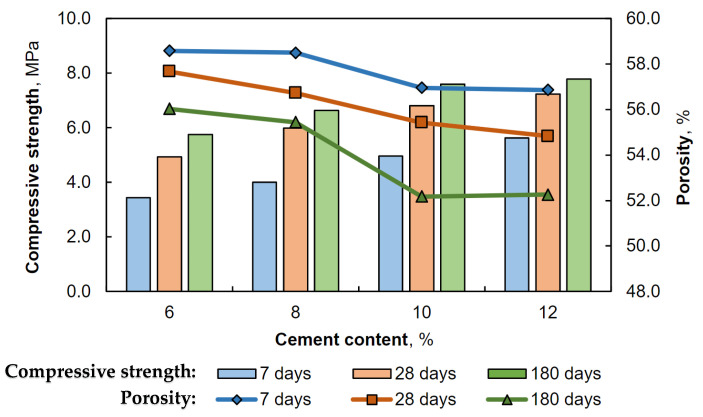
Relationship between compressive strength and porosity of gypsum–cement mortars on the 7th, 28th, and 180th day of conditioning in increased relative humidity (>95%).

The relationship between porosity and compressive strength was observed for the tested gypsum–cement plasters. Plasters with a higher cement content (30G/12C, 30G/10C) exhibited lower porosity and higher compressive strength. This relationship is summarized graphically in Figure 5.

**Figure 6:**
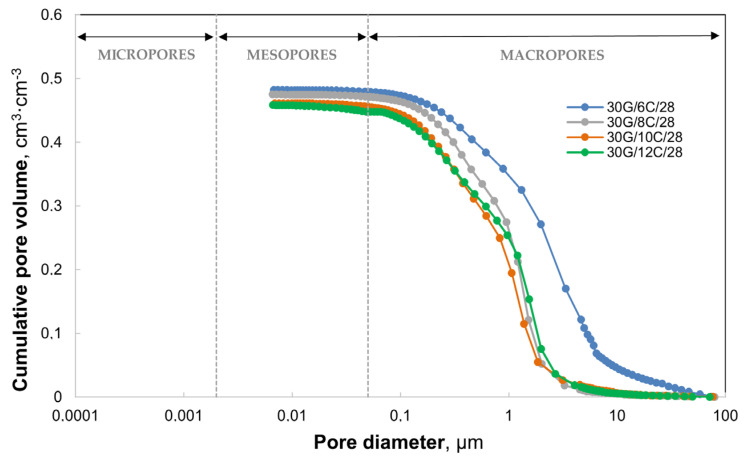
Cumulative pore volume in gypsum–cement plasters after 28 days of seasoning in increased relative humidity (>95%). The broken lines indicate the classification of pores according to the International Union of Pure and Applied Chemistry (IUPAC).

In gypsum–cement plaster samples after 28 days of hydration, the total volume of pores with a diameter of less than 100 µm ranges from 0.456 to 0.481 cm^3^/cm^3^ (Figure 6). The test results presented in Figure 6 indicate that the total pore volume decreases with the increase in the amount of cement in the plasters. The most significant differences in porosity occurred between the plaster with the addition of 6% cement and the remaining plasters containing 8, 10, and 12% cement, showing a similar relationship between the total pore volume and their diameter. After 28 days, the 30G/6C plaster exhibits the highest content of capillary pores (macro capillaries) compared to the other plasters. Plasters 30G/8C, 30G/10C, and 30G/12C show similar porosity in the pore range from 1 to 10 µm, while in the pore range below 1 µm, the 30G/10C and 30G/12C plasters showed lower porosity than the 30G/8C plaster.

**Figure 7:**
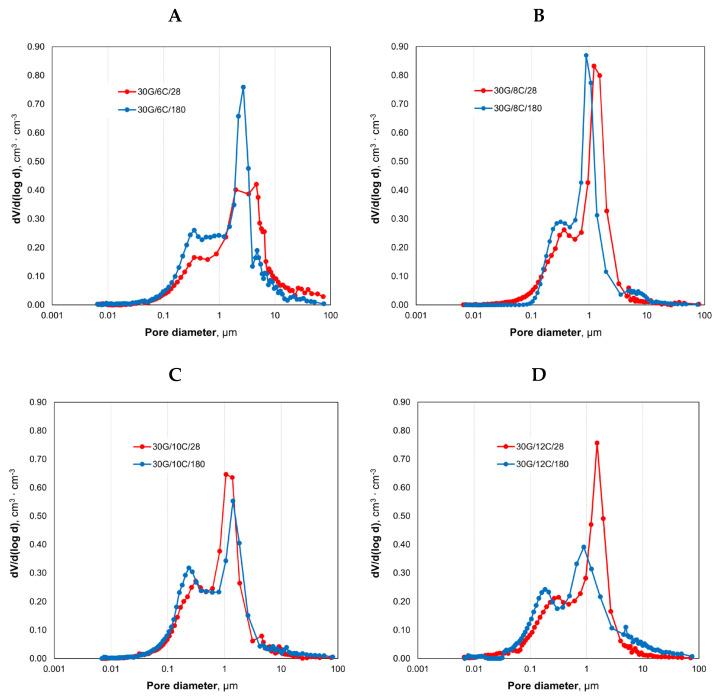
Pore size distribution between 28 and 180 days of seasoning in increased relative humidity (>95%): (**A**)—30G/6C plaster, (**B**)—30G/8C plaster, (**C**)—30G/10C plaster, (**D**)—30G/12C plaster.

Figure 7A–D show bimodal pore size distributions for gypsum–cement plasters after 28 and 180 days of seasoning. After the 180th day of conditioning, all plasters exhibited a shift in pore size distributions towards pores with smaller diameters, as presented in Figure 7 and Table 3.

In all plaster samples (Table 3), after 180 days of seasoning, a decrease in the content of macropores above 1 µm and an increase in the share of macropores in the range of 0.1–1.0 µm was observed concerning their content in the 28th day of hydration.

The content of the pores with a diameter below 0.1 µm (macropores 0.05–0.1 µm, mesopores 0.002–0.05 µm and micropores below 0.002 µm according to IUPAC) between 28th and 180th day of seasoning did not change in the 30G/6C plaster, decreased in the 30G/8C sample, and increased in 30G/10C and 30G/12C plasters. The highest pore content, with a diameter lower than 0.1 μm was found for the plaster with 12% cement content after 180 days and equal to 19%.

The demonstrated changes in the total pore volume and pore size distributions of plasters after 28 days and 180 days may result from the sealing of the microstructure, which is related to the formation of C-S-H and other hydration products (ettringite, thaumasite) [46,47,48].

Table 4 presents parameters describing the structure of the pore system in the tested plasters. According to Diamond [49], the threshold diameter measures the effective diameter of the continuous pores in the paste.

Due to deviations of the pore geometry in mineral binder pastes from the model used in mercury porosimetry, the threshold diameter serves as an adequate measure of the material’s permeability. The second parameter of the porosimetric curve that Diamond [49] points to as a valuable measure describing the pore system is the total volume of injected mercury. One can notice that the plaster containing 6% Portland cement has a larger threshold diameter than plasters with a higher cement content, for which changes in the threshold diameter value are no longer significant.

Generally, one can say that from the point of view of the threshold diameter, the differences between individual plasters are within relatively narrow limits. These values fall between the threshold diameter values for cement slurries and gypsum slurries. Diamond [49] provides exemplary porosimetric curves of cement slurries after 2 and 320 days of hydration. The threshold diameters of such slurries are in the range of 0.8–1.0 µm for *w*/*c* = 0.6 and 0.08–0.10 µm for the *w*/*c* ratio = 0.4. Thisconfirms results of extensive research conducted by Cook and Hover [50]. Unlike porosimetric tests of cement composites, tests of the porosity structure using the MIP method for gypsum slurries are much less common. According to Svorová-Pawełkowicz et al. [51], the threshold diameter of hardened gypsum slurry made of high-purity bassanite with a *w*/*g* ratio = 0.38 was approximately 9 µm. This means that gypsum–cement materials, from the point of view of the threshold diameter, are between gypsum and cement composites. However, the threshold diameters are closer in order of magnitude to those of gypsum materials. This shows that relatively large gypsum crystals, which crystallize first, still shape the basic microstructure.

The total volume of injected mercury, apart from minor fluctuations for samples aged 180 days, decreases with the amount of cement added. It indicates a reduction in open porosity, a good prognosis for the material’s durability under unfavorable environmental conditions.

### 3.4. Scanning Electron Microscopy (SEM)

**Figure 8:**
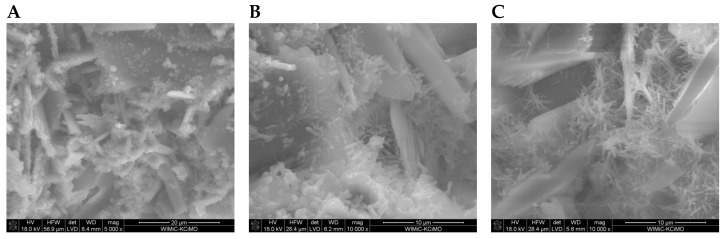
Microstructure of 30G/6C plaster after (**A**) 7, (**B**) 28, and (**C**) 180 days of conditioning in increased relative humidity (RH > 95%). (**A**)—magnification 5000; (**B**,**C**)—magnification 10,000.

**Figure 9:**
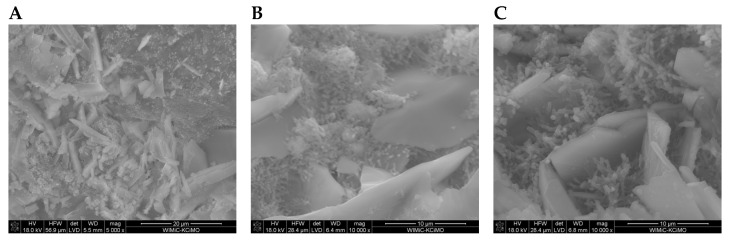
Microstructure of 30G/8C plaster after (**A**) 7, (**B**) 28, and (**C**) 180 days of conditioning in increased relative humidity (RH > 95%). (**A**)—magnification 5000; (**B**,**C**)—magnification 10,000.

**Figure 10:**
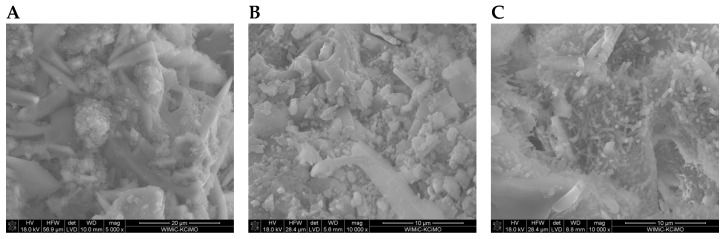
Microstructure of 30G/10C plaster after (**A**) 7, (**B**) 28, and (**C**) 180 days of conditioning in increased relative humidity (RH > 95%). (**A**)—magnification 5000; (**B**,**C**)—magnification 10,000.

**Figure 11:**
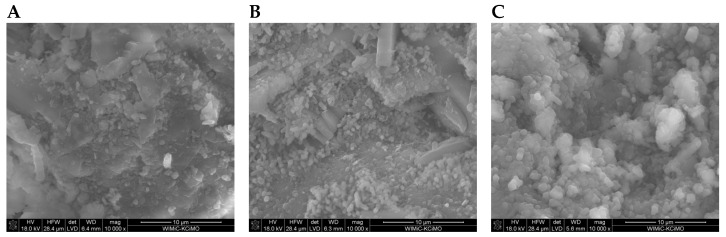
Microstructure of 30G/12C plaster after (**A**) 7, (**B**) 28, and (**C**) 180 days of conditioning in increased relative humidity (RH > 95%). (**A**)—magnification 5000; (**B**,**C**)—magnification 10,000.

SEM images of the microstructure of plasters (Figure 8, Figure 9, Figure 10 and Figure 11) showed significant differences depending on the amount of cement added.

In the SEM photos of plasters on the 7th day of hydration with 6%, 8%, and 10% cement (Figure 8A, Figure 9A and Figure 10A), gypsum crystals and a small amount of the C-S-H phase are visible. The SEM image of plaster 30G/12C (Figure 11A), containing the highest amount of cement, shows a higher compaction degree than the other three plasters. The decrease in porosity that occurs with the maturation of the plaster samples (28 and 180 days), leading to a more compact structure, was attributed to the increasing amount of hydration products, mainly the C-S-H phase, which grow on the gypsum crystals and fill the free spaces between them (Figure 8B,C, Figure 9B,C and Figure 11B,C).

**Figure 12:**
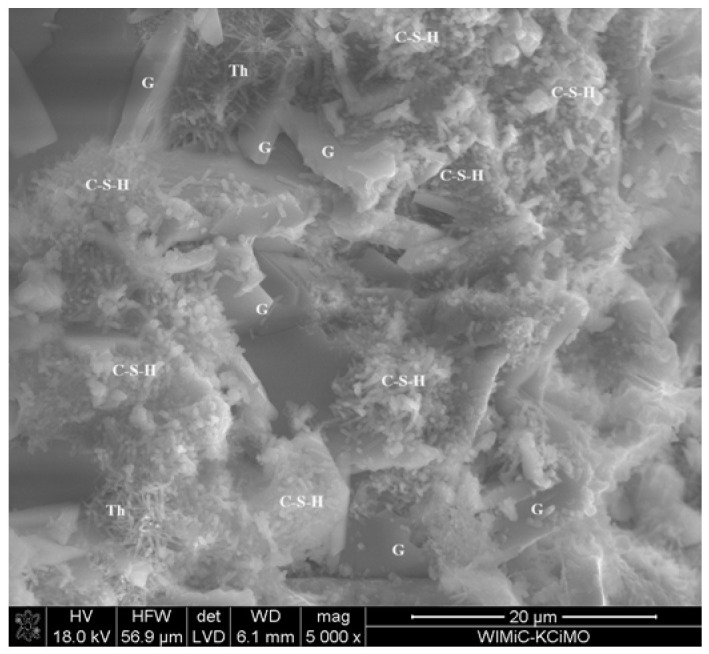
SEM image of the microstructure of 30G/10C plaster after 180 days of conditioning in increased relative humidity (RH > 95%).

**Figure 13:**
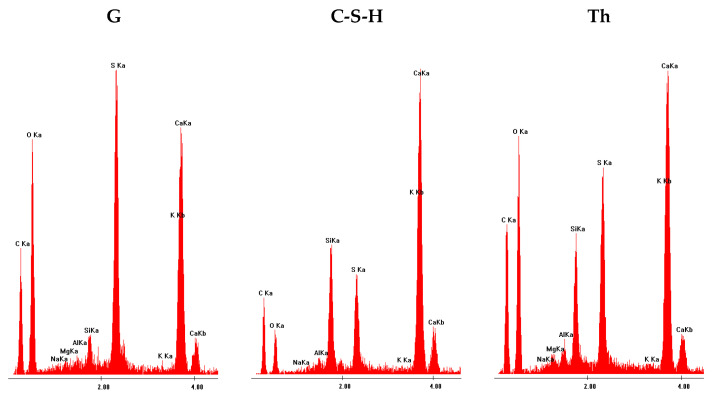
EDS spectra of characteristic structures of gypsum (G), calcium silicates hydrates (C-S-H) and thaumasite (Th).

**Figure 14:**
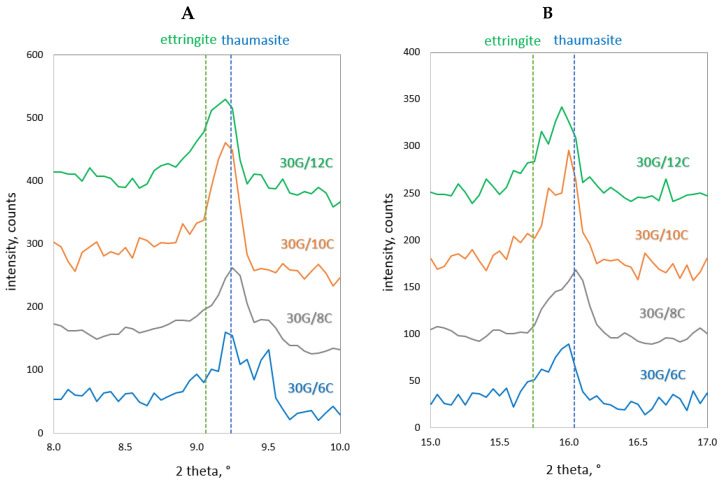
X-ray diffraction patterns of gypsum–cement plasters cured at increased humidity (RH > 95%) for 28 days; (**A**) 2θ = 8–10°, (**B**) 2θ = 15–17°.

**Figure 15:**
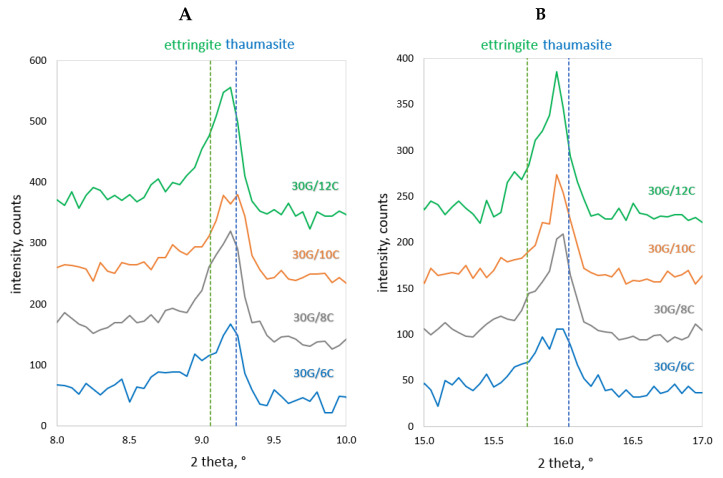
X-ray diffraction patterns of gypsum–cement plasters cured at increased humidity (RH > 95%) for 180 days; (**A**) 2θ = 8–10°, (**B**) 2θ = 15–17°.

Figure 12 shows an example SEM image of the 30G/10C plaster after 180 days of seasoning, with the characteristic structures of gypsum (G), the C-S-H phase (C-S-H) and thaumasite (Th) [25] marked, and the EDS spectra recorded for these structures (Figure 13), confirming the presence of the phases mentioned above. XRD diffractograms confirming the presence of thaumasite and/or mixed crystals of ettringite and thaumasite at days 28 and 180 are provided in Figure 14 and Figure 15. Detailed explanations of the formation of these phases were provided in an earlier publication by the authors [25].

### 3.5. Visual Assessment of the Durability of Plaster Bars on Individual Days of Storage

**Figure 16:**
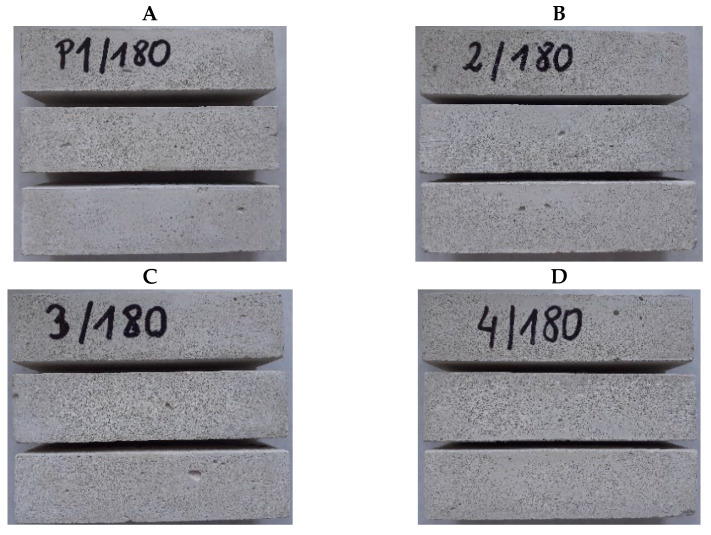
Bars of gypsum–cement-based plasters after 180 days of seasoning in conditions of increased relative humidity (RH > 95%) and drying to a constant mass at 45 °C; (**A**) P1/180—plaster 30G/6C; (**B**) 2/180—plaster 30G/8C; (**C**) 3/180—plaster 30G/10C; (**D**) 4/180—plaster 30G/12C.

Figure 16 shows photos of gypsum–cement plaster bars taken after 180 days of seasoning.

Despite the confirmation of the formation of ettringite and thaumasite in the plasters during prolonged conditioning at increased relative humidity [25], no damage was found inside (SEM images) or on the surface of the bars (Figure 16). Characteristic signs of damage resulting from thaumasite crystallization could include, for example, peeling of the surface of hardened composites, cracks or degradation of structural defects, and their transformation into a “white, non-binding mass” [52]. After 180 days of conditioning in increased humidity conditions, plaster samples exhibited a stable and durable structure, as confirmed by the mechanical strength results (Table 2, Figure 3).

## 4. Conclusions

(1) Adding Portland cement increases the strength of gypsum–cement plasters seasoned in conditions of increased relative humidity (>95%). Flexural and compressive strengths increase with the hydration progression to the 180th day.
(2) The bulk density of gypsum–cement plasters increases with the increase in the cement addition in the plaster and the seasoning time.
(3) The decrease in porosity of the tested plasters is the result of the formation of the C-S-H phase as a result of the hydration process of silicate phases and probably the crystallization of ettringite, thaumasite or mixed ettringite-thaumasite crystals. Reducing the porosity of plasters increases their mechanical strength.
(4) After 28 and 180 days of seasoning, the tested plasters exhibited a bimodal pore size distribution. During this period, changes in the pore size distribution occurred, characterized by an increase in pore numbers with smaller diameters. This change is attributed to the sealing of the microstructure by the formation of the C-S-H phase and other hydration products.
(5) All plasters exhibited increasingly compact microstructures with longer curing times and higher amounts of added cement. The most compact structure characterizes a sample containing 12% cement after 180 days of seasoning.
(6) The results have shown that new plasters based on a mixture of gypsum and cement binders fulfills requirements of EN 13279:1-2008 for lightweight gypsum-based plasters (category B5) but also for cement plastering mortars (categories CSII and CSIII). According to EN 998-1:2010 [53], plasters of these categories should meet compressive strength requirements from 1.5 to 5.0 N/mm^2^ (CSII) and from 3.5 to 7.5 N/mm^2^ (CSIII). This means that using new gypsum–cement plaster to replace both commonly used plasters is possible with a significant reduction in gypsum and cement content. The solution proposed in the paper of reducing the amount of cement in mortars will also reduce the amount of greenhouse gases (mainly CO_2_) from cement production.

## Figures and Tables

**Table 1:** Composition of gypsum–cement plasters.

Components	Type of Plaster			
	30G/6C	30G/8C	30G/10C	30G/12C
	%			
Gypsum	30.00	30.00	30.00	30.00
CEM I 42,5R	6.00	8.00	10.00	12.00
Quartz sand (0.1–0.5 mm)	48.59	46.59	44.59	42.59
Limestone powder (0.0–0.2 mm)	10.00	10.00	10.00	10.00
Hydrated lime	2.00	2.00	2.00	2.00
Expanded perlite (0.0–2.0 mm)	3.00	3.00	3,00	3.00
Additives	0.41	0.41	0.41	0.41
Total	100.00	100.00	100.00	100.00

**Table 2:** Specific and bulk density, porosity, and mechanical strength of gypsum–cement plasters conditioning in increased relative humidity (>95%).

Sample	Age Days	Specific Density g/cm^3^	Standard Deviation for Specific Density	Level of Significance *** (*p* ≤ 0.001)	Bulk Density g/cm^3^	Porosity %	Mechanical Strength	
							Flexural MPa	Compressive MPa
30G/6C	7	2.475	0.003		1.025	58.56	1.48	3.44
	28	2.454	0.003		1.039	57.69	1.76	4.93
	180	2.430	0.003		1.068	56.03	1.76	5.75
30G/8C	7	2.474	0.003		1.027	58.50	1.58	4.01
	28	2.422	0.002	***	1.048	56.73	2.03	6.00
	180	2.406	0.002	***	1.072	55.45	2.13	6.63
30G/10C	7	2.429	0.002	***	1.045	56.96	1.75	4.96
	28	2.384	0.002	***	1.063	55.41	2.31	6.80
	180	2.289	0.002	***	1.094	52.18	2.46	7.60
30G/12C	7	2.430	0.002	***	1.048	56.86	2.02	5.63
	28	2.381	0.003	***	1.075	54.83	2.51	7.24
	180	2.303	0.003	***	1.099	52.26	2.62	7.79

**Table 3:** Pore diameter distribution for plasters after 28 and 180 days of seasoning in increased relative humidity (>95%).

Sample	Age Days	Content of Pores % by Volume		
		>1.0 µm	0.1–1.0 µm	<0.1 µm
30G/6C	28	74.3	21.7	4.0
	180	60.5	35.5	4.0
30G/8C	28	45.8	49.2	5.0
	180	33.3	66.3	0.4
30G/10C	28	40.4	49.7	9.9
	180	36.0	53.8	10.2
30G/12C	28	42.5	45.1	12.4
	180	32.1	48.9	19.0

**Table 4:** Mercury Intrusion Porosimetry (MIP) results.

Sample	Age	Cement Content	Threshold Diameter	Total Mercury Volume	Calculated Porosity
	Days	%	μm	cm^3^/cm^3^	% by Volume
30G/6C	28	6	6.8	0.481	48.1
30G/8C		8	4.5	0.473	47.3
30G/10C		10	4.5	0.463	46.3
30G/12C		12	4.1	0.456	45.6
30G/6C	180	6	4.0	0.495	49.5
30G/8C		8	3.5	0.449	44.9
30G/10C		10	4.2	0.479	47.9
30G/12C		12	4.6	0.435	43.5

## Data Availability

Data are contained within the article.
